# The effect of *Aspergillus oryzae* fermentation product on production parameters, rumen environment, and fiber degradability of Jersey cows grazing ryegrass-dominant pasture

**DOI:** 10.1093/jas/skaf282

**Published:** 2025-08-20

**Authors:** Cherise Basson, Lobke Steyn, Robin Meeske, Fernando Bargo

**Affiliations:** Department of Animal Sciences, Stellenbosch University, Stellenbosch, South Africa7600; Department of Animal Sciences, Stellenbosch University, Stellenbosch, South Africa7600; Department of Animal Sciences, Stellenbosch University, Stellenbosch, South Africa7600; Western Cape Department of Agriculture, Outeniqua Research Farm, George, South Africa6529; Department of Business and Inovation, Biozyme Inc., St. Joseph, MO 64504, USA

**Keywords:** *Aspergillus oryzae*, fiber, milk fat, pasture-based

## Abstract

*Aspergillus oryzae* fermentation product (AOFP; Amaferm—Biozyme Inc., St. Joseph, MO, USA) is a feed additive produced from a dried fermentation extract of the fungus *Aspergillus oryzae* NRRL458. Previous research on AOFP supplementation were done on total mixed rations and in vitro systems, with research on pasture-based systems lacking. The aim of the study was to evaluate the impact of AOFP on milk production, milk composition, rumen environment, fiber degradation, and dry matter intake (DMI) in Jersey cows grazing ryegrass pasture. Treatments in this study were as follows: Control—cows received 6 kg per cow per day (as-fed) of a pelleted dairy concentrate (159.6 g.kg^-1^ DM crude protein and 12.3 MJ metabolizable energy.kg^-1^ DM), and AOFP treatment—cows received 6 kg per cow per day (as-fed) of the same dairy concentrate with AOFP mixed in at 0.5 g.kg^-1^ (3 g.cow^-1^.d^-1^; as-fed). In the production study, 34 (*n* = 17) early-to-mid lactation cows were selected, blocked, and randomly allocated to treatments. From this group, 20 cows (*n* = 10) were randomly selected for the DMI study. For the rumen Study, 6 rumen-cannulated cows were used in a crossover design with 2 treatments and 2 periods. Data collected for the production study included daily milk production and milk composition. DMI was determined using the TiO_2_ method. During the study of the rumen, rumen pH was recorded for 3 continuous days. Rumen fluid was sampled and analyzed for volatile fatty acid (**VFA**) and ammonia nitrogen concentrations (**NH**_**3**_**-N**), and an *in sacco* dacron bag study was performed to determine dry matter (**DMd**) and neutral detergent fiber (NDF) disappearance (**NDFd**). Milk production and composition did not differ between treatment groups (*P* ≥ 0.05). Milk fat content tended (*P* ≤ 0.10) to be 2.3 g.kg^-1^ greater in the AOFP treatment group. DMI was similar (*P* ≥ 0.05) between treatment groups. Average rumen pH was lower (*P* < 0.05) for cows on the AOFP treatment but remained within an optimal range for rumen microbial function. Rumen VFA and NH_3_-N concentrations did not differ (*P* ≥ 0.05). The *in sacco* dry matter and NDF disappearance were not affected (*P* ≥ 0.05) by AOFP supplementation. To conclude, supplementation of AOFP to cows grazing ryegrass did not affect milk production but tended to increase milk fat content. Rumen parameters and degradability of ryegrass were not affected by AOFP supplementation.

## Teaser

When pasture-based dairy cows were supplemented with *Aspergillus oryzae* fermentation product, milk production remained unchanged, but AOFP tended to increase milk fat content. Fiber degradation and dry matter intake were similar between treatments and rumen pH remained within the optimal range for proper rumen function.

## Graphical Abstract

**Graphical Abstract:**
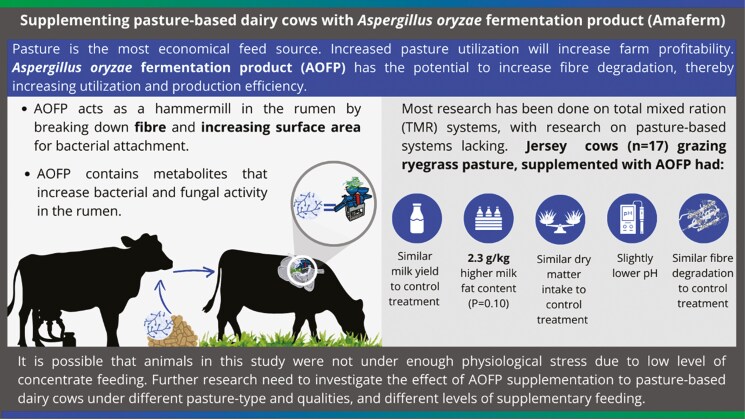

## Introduction

Feed costs contribute largely to the input costs of dairy production systems. Input costs of pasture-based systems are typically lower compared to total mixed ration (**TMR**) dairy systems, primarily because pasture is the most economical feed source (Bargo et al., 2003). In pasture-based systems, energy becomes the first limiting factor for production (Nisbet & Martin, 1990). Consequently, farmers supplement with concentrates to meet the cow’s metabolizable energy (ME) requirements. Depending on the levels of concentrate feeding, the rumen environment can be negatively affected, leading to decreased pH and reduced fiber degradability (Nisbet & Martin, 1990; Van Soest et al., 1991; Faniyi et al., 2019). Maximizing pasture utilization in pasture-based systems can lower input costs, enhance profitability, and promote a healthier rumen environment (Sucu et al., 2019).

Feed additives such as prebiotics, probiotics and postbiotics are used in livestock production systems to enhance dairy cow health, digestion, and milk production (El Jeni et al., 2024). While antibiotic-based feed additives have been used for many years, consumer concerns over residues in animal products have led to the development of alternative products (Kholif et al., 2024).

*Aspergillus oryzae* fermentation product (**AOFP**; Amaferm—BioZyme Inc., St. Joseph, MO, USA) is a prebiotic feed additive produced from the fermentation extract of a selected strain of the fungus *Aspergillus oryzae* (NRRL458), which is freeze-dried on bran (Zhang et al., 2022). *Aspergillus oryzae* produces metabolites, which enhance the activity of lactate-utilizing bacteria, *Megasphaera elsdenii* and *Selenomonas ruminantium* (Bercovits et al., 1990; Nisbet & Martin, 1990; Sallam et al., 2020; Kholif et al., 2024). This increases lactate utilization in the rumen, limiting build-up and mitigating the post-concentrate feeding ruminal pH drop, which is commonly observed in pasture-based dairy cows (Kholif et al., 2024). The more stable rumen pH increases cellulolytic bacterial activity and improves fiber degradation (Nisbet & Martin, 1990).

Fungi in the rumen also utilize the metabolites in AOFP (Orpin, 1977; Borneman et al., 1992; Varel et al., 1993; Wubah, 2004), resulting in increased growth rate and rhizoid branching, enhancing the activity of fungi. This results in increased breakage of the lignin-hemicellulose bonds and increased production of fiber-degrading enzymes that break down hemicellulose and cellulose (Orpin, 1977; Borneman et al., 1992; Varel et al., 1993; Chang et al., 1999; Schmidt et al., 2004 ; Wubah, 2004). Breakage of the lignin-hemicellulose bonds increases the accessibility of plant material to bacterial species and increases the surface area for bacterial attachment (Orpin, 1977; Borneman et al., 1992; Varel et al., 1993; Wubah, 2004). In an in vitro study by Nisbet & Martin (1990), it was found that AOFP increased the production of acetate, propionate, and the total volatile fatty acids (**VFA**) in the rumen, thus altering the VFA profile. This is possibly due to increased fungal and bacterial activity in the rumen. The change in the VFA profile affects milk yield and milk composition in dairy cows, especially considering that acetate is the precursor for fat biosynthesis (Sallam et al., 2020).

Cantet et al. (2019) performed a meta-analysis on dairy cows in TMR-based systems, which concluded that AOFP improves fiber degradation and increases dry matter intake (**DMI**) and fat-corrected milk yield (**FCM**). In early-lactation cows, AOFP supplemented at 15 g.d^-1^, has been linked to improved energy balances, as evidenced by increased milk yield and reduced plasma non-esterified fatty acid concentrations, despite no differences in body weight change (Sucu et al., 2019; Cantet et al., 2025). Dairy cows experiencing heat stress had lower inflammatory markers and increased milk yield when supplemented with AOFP (Kaufman et al., 2021). Similar anti-inflammatory and gut health-enhancing effects have been reported in non-ruminant species (Chuang et al., 2019; Dawood et al., 2019). A recent study by Zhang et al. (2022) showed that AOFP supplementation in cows resulted in decreased lactate and lipopolysaccharide concentrations in the rumen, and significantly altered hindgut pH, VFA profiles, and microbial populations. These findings point to a broader effect of AOFP that may include enhanced hindgut fermentation, reduced systemic inflammation, and improved nutrient partitioning during metabolically challenged periods (Cantet et al., 2025).

While AOFP has been extensively studied in vitro and on cows in TMR-based systems, little is known about its effects in pasture-based dairy systems. The primary objective of this study was to evaluate the effect of supplementing Jersey cows grazing perennial ryegrass pasture with AOFP during spring on milk production and composition, and DMI. Secondary objectives were to assess the effect of AOFP on the rumen environment, including fiber degradability, rumen pH, rumen VFA, and ammonia nitrogen (**NH**_**3**_**-N**) concentrations. It was hypothesized that supplementing grazing cows with AOFP would enhance fiber degradation, leading to increased DMI, increased milk production, and increased milk fat content. This study aimed to provide insight into the potential of AOFP as a nutritional strategy to optimize the performance of pasture-based dairy cows.

## Materials and Methods

This study was conducted at Outeniqua Research Farm, George, South Africa, during Spring 2023. Ethical approval was obtained from the Research Ethics Committee for Animal Care and Use in Research (ACU-2023-27577).

### Animals, experimental design, and treatments

The study comprised both a production study and a rumen evaluation study. The production study was a randomized block design with 2 treatments. Thirty-four multiparous early to mid-lactation Jersey cows (*n* = 17) were selected from a group of 60 cows using milk production and milk composition data (2 composite milk samples for each cow) that were collected during a 2-wk pretrial period. Cows were then blocked and randomly allocated to the 2 treatments based on milk production, milk composition, days in milk, and lactation number (**Table 1**). Body condition score and live weight were not included in the blocking criteria, as blocking on more than 3 variables complicates accurate treatment allocation in dairy cow trials. However, body condition scoring and live weight were confirmed to be statistically similar between treatment groups at the onset of the trial. Cows were ear-tagged with different color tags to differentiate between treatments. Cows were subjected to a 3-wk adaptation period followed by a 6-wk measurement period.

**Table 1.** Pretrial milk yield, milk composition, lactation number, and days in milk (DIM; mean ± SD) of the Jersey cows used in the study (*n* = 17)

Parameter	Treatments^1^	
	Control	AOFP
Milk yield, kg.cow^-1^.d^-1^	20.2 ± 2.20	20.3 ± 1.86
4% FCM yield, kg.cow^-1^.d^-1^	21.9 ± 2.48	22.1 ± 2.23
Milk fat, g.kg^-1^	45.6 ± 0.590	45.9 ± 0.574
Milk protein, g.kg^-1^	36.6 ± 0.252	36.2 ± 0.324
Lactation number	4.76 ± 1.786	4.06 ± 1.60
DIM	108 ± 58.6	116 ± 66.9

FCM, fat-corrected milk; DIM, days in milk.

^1^Control—6 kg.cow^-1^.d^-1^ standard dairy concentrate; AOFP—6 kg.cow^-1^.d^-1^ standard dairy concentrate containing 0.5 kg.ton^-1^*Aspergillus oryzae* fermentation product (AOFP; Amaferm).

Treatments were as follows:

1. Control—6 kg.cow^-1^.d^-1^ standard dairy concentrate.
2. AOFP treatment—6 kg.cow^-1^.d^-1^ standard dairy concentrate + Amaferm (**AOFP**; BioZyme Inc., St. Joseph, MO, USA) at 0.5 kg.ton^-1^ (3 g.cow^-1^.d^-1^).

The rumen study included 6 rumen-cannulated cows in a 2-by-2 crossover design, with 2 treatments and 2 periods. Cows were allowed a 21-d adaptation period before and between each 7-d measurement period.

Cows grazed ryegrass-dominant pasture under sprinkler irrigation and received 6 kg concentrate per cow per day (as-fed) divided between 2 milkings (06:00, 14:00) in the milking parlor. The concentrate was weighed into 3 kg bags and manually emptied into the individual feeding troughs in the parlor before each milking. The ingredients and calculated nutrient composition of the 2 concentrate treatments are shown in **Table 2**. All ingredients were sourced in South Africa in 2023. Concentrates were formulated for 160 g crude protein (**CP**).kg^-1^ DM and 12.3 MJ ME.kg^-1^ DM. *AOFP* was included at a rate of 0.5 kg per metric ton of concentrate, equating to a daily intake of 3 g. cow^-1^.d^-1^, based on the 6 kg of concentrate fed per cow per day. This dosage aligns with the manufacturer’s recommendation for lactating dairy cows (Biozyme, n.d.). Although most published studies on AOFP have been conducted under TMR systems, the same recommended dosage was used to assess the product’s efficacy in pasture-based systems without exceeding safe, practical, or supplementation limits.

**Table 2.** Ingredients and calculated nutrient composition (g.kg^-1^ DM) of the 2 concentrates fed at 6 kg (as-fed) per cow per day (*n* = 17)

Parameter	Treatments^3^	
	Control	AOFP
**Ingredients, g.kg** ^**-1**^ **DM** ^1^		
White maize fine	498	498
Soya oilcake	152.7	153.4
Wheat Bran	145.5	142.2
Hominy Chop	126	128
Molasses	37.0	37.0
Limestone	30.6	30.6
Salt	4.54	4.54
Magnesium oxide	1.29	1.32
Vitamin premix	4.33	4.33
AOFP	0	0.54
**Calculated nutrient specifications, g.kg** ^**-1**^ **DM** ^2^		
DM	877	877
CP	160	160
ME, MJ.kg^-1^	12.3	12.3
NDF	161	160
Starch	461	461
Fat	35.6	35.6
Ca	11.4	11.4
P	5.01	5.01
Mg	4.56	4.56

^1^AOFP—*Aspergillus oryzae* fermentation product, Vitamin premix – 4 kg pre-mixed pack (Vit A: 6,000,000 IU, Vit D: 1,000,000 IU, Vit E: 8,000 IU, Mn 50g, Zn 100 g, Cu 20 g, I 1.7g, Se 0.3 g and carrier Dolomite: 440g).

^2^DM, Dry matter; CP, Crude protein; ME, Metabolizable energy; NDF, Neutral detergent fiber; Ca, Calcium; P, Phosphor; Mg, Magnesium.

^3^Control—6 kg.cow^-1^.d^-1^ standard dairy concentrate; AOFP—6 kg.cow^-1^.d^-1^ standard dairy concentrate containing 0.5 kg.ton^-1^*Aspergillus oryzae* fermentation product (AOFP; Amaferm).

Pasture consisted of established kikuyu (*Cenchrus clandestinus*), over-sown with perennial ryegrass *(Lolium perenne*; Cultivar: 15 kg Viscount and 10 kg Platform) in Autumn 2023. Pasture was fertilized after each grazing with 100 kg.ha^-1^ of 28% nitrogen limestone ammonium nitrate. Ryegrass was grazed at a 2-and-a-half to 3-leaf stage. Strip grazing was implemented, and cows were moved to fresh pastures after each milking. A rising plate meter (**RPM**) was used to measure pre- and post-grazing pasture heights, and a regression equation obtained from Van Wyngaard (2018) was used to estimate pasture yield and adjust pasture allocation: Y = (102.99 × H) − 260.79; R2 = 0.7282. During Spring in the Southern Cape of South Africa, a “spring flush” occurs, making pasture management challenging. Efficient utilization of pasture during this period requires adjusting allocations based on leaf stage, RPM heights, and direct observation. Cows from both treatment groups grazed together as one group in the same paddock daily under controlled allocation.

Concentrate and pasture samples were collected weekly for proximate analysis. Pasture and concentrate samples were dried in a Labcon oven at 60 °C for 72 h and milled through a Wiley mill to pass through a 1 mm sieve. Samples were analyzed for DM (AOAC, 2002; Method 934.01), organic matter (OM; AOAC, 2002; Method 942.05), neutral detergent fiber (**NDF**; Goering & Van Soest, 1970), acid detergent fiber (**ADF**; Goering & Van Soest, 1970), CP (Leco), ether extract (**EE**; AOAC, 2002; Method 920.39), gross energy (**GE**), and macro and micro minerals (ALASA, 2007; Dry Ashing method 6.1.1). Concentrate and pasture in vitro dry matter degradability (**IVDMD**) was determined using an ANKOM Daisy ^II^ Incubator (Ankom Technology Corp, NY, USA) according to the official ANKOM procedures. Concentrate ME was calculated according to Robinson et al. (2004): **ME (MJ.kg**^**-1**^**) (DM) = GE × IVDMD × 0.82**. Pasture ME was calculated using the MILK 2016 Excel spreadsheet developed by Undersander et al. (2016).

### Production study

Cows were weighed (Tru-Test Ezi-Weigh 2, serial no. 542707) and body condition was scored twice on 2 consecutive days at the start and end of the trial. Body condition scoring was performed by a trained technician according to the methods described in Wildman et al. (1982).

Daily milk production was recorded using the Afimilk management system. A 20-point Waikato swing-over milking machine with electronic meters was used to milk the cows. Milk samples were taken weekly, and a composite sample was obtained by combining 16 mL of morning milk and 8 mL of afternoon milk. The composite milk samples were sent to Merieux Nutriscience Pty (Ltd), where a Milkoscan FT 6,000 machine (Foss Electric, Denmark) was used to analyze the samples for fat, protein, somatic cell count, and milk urea nitrogen (**MUN**) content. The 4% fat-corrected milk (**FCM**) yield and energy-corrected milk (**ECM**) yield were calculated using equations from Gaines & Davidson (1923) and Sjuanja et al. (1990), respectively.

### Dry matter intake study

Pasture DMI was estimated using titanium dioxide (**TiO**_**2**_) as an external marker and indigestible NDF (**iNDF**) as an internal marker. Ten cows per treatment (*n* = 10) were randomly selected from the production study groups. Cows were dosed with gelatin capsules containing 3 g of TiO_2 (_Titanium (IV) oxide 14027; Extra pure, 99% to 100.5%; M = 79.87 g.mol^-1^; CAS-No: 13463-67-7; https://www.sigmaaldrich.com/life science), after each milking, for 10 consecutive days. Fecal samples were collected twice daily during the last 5 d of dosing. Fecal samples were dried in a draft oven at 80 °C until all samples were dry. Samples were milled using a Willey mill to pass a 1 mm sieve and stored for analysis. Samples were analyzed for TiO_2_ concentration by Bemlab (https://www.bemlab.co.za/). The iNDF of the concentrate, pasture, and fecal samples were determined according to the methods described by Valentine et al. (2018). Fecal excretion and pasture DMI were calculated using formulas from De Souza et al. (2015):


$$\begin{array}{l} Total\;fecal\;excretion\;\left(kg.day^{\text{-}1}\right)\;=\frac{Amount\;TiO_{2}\;dosed\;(g.day^{-1})}{Concentration\;TiO_{2}\;in\;feces\;(g.kg^{-1})\;} \end{array}$$

$$\begin{array}{l} Pasture\;DMI\;\left(kg.{day}^{\text{-}1}\right)\;=\frac{\begin{array}{c} \left[(Total\;fecal\;excretion\;(kg.day^{-1})\;\;\times \;iNDF\;feces\;concentration\;(kg.kg^{-1})\;-iNDF\;intake\;from\;concentrate\;(kg.day^{-1})\right] \end{array}}{iNDF\;forage\;(kg.kg^{-1})} \end{array}$$

Feed efficiency was calculated as the ratio of ECM to total DMI, expressed as kg ECM per kg of DMI.

### Rumen study

**Figure 1.**
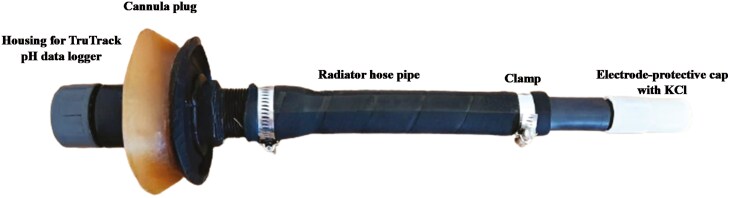
The TruTrack pH Data Loggers (Model pH-HR mark 4, Intech Instruments LTD, NZ) were used to measure diurnal rumen pH over a continuous 72-h period.

The rumen pH of rumen-cannulated cows was recorded for 72 h using an indwelling TruTrack pH data logger (Model pH-HR mark 4, Intech Instruments, LTD, New Zealand) housed in a radiator hose fitted to a cannula plug (**Figure 1**). The pH loggers were calibrated at pH 4.0 and pH 9.0 and assessed at pH 7.0 before pH measurement.

**Figure 2.**
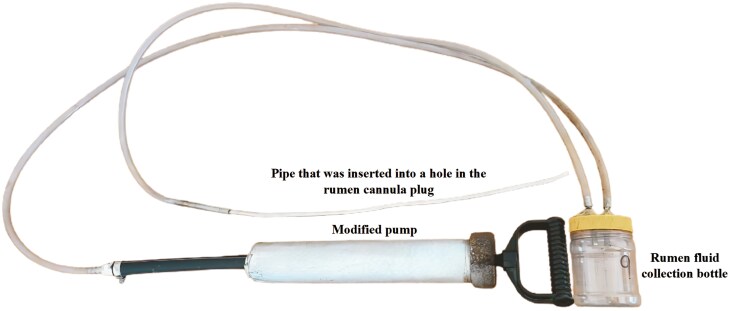
The pump was modified to function as a suction device, creating a vacuum within the system, facilitating rumen fluid collection.

Rumen fluid samples were collected at 3 time intervals (06:00; 14:00; 22:00) using a modified pump (**Figure 2**). Directly after collection, the pH of the rumen samples was measured using a WTW pH 340i meter and WTW Sentix®41 pH electrode. Rumen fluid was filtered through a double-layered cheesecloth, poured into airtight containers, and frozen for later analysis. Samples were analyzed for VFAs (Siegfried et al., 1984) and rumen ammonia nitrogen (**NH**_**3**_**-N**; Broderick & Kang, 1980).

An *in sacco* dacron bag study was performed according to Cruywagen (2006) to determine the rate and extent of DM and NDF degradation of ryegrass-dominant pasture at 6-, 18-, and 30-h intervals. After incubation, bags (53 µm pore size and 10 cm × 20 cm) were rinsed with clean water and frozen for further processing. When collection was complete, all bags were thawed, washed in a washing machine for 5 gentle cycles, each 3 min long (clean water used for each cycle), and dried in a Labcon oven at 60 °C for 72 h. Samples were weighed to calculate the extent of DM degradability and then milled with a Wiley mill to pass through a 1 mm sieve. Samples were pooled per cow, for each removal time and period. Samples were analyzed for NDF with heat-stable alpha-amylase (aNDF) content (Analytical Methods, Fiber Analyzer A200 | ANKOM Technology) to determine the extent of NDF degradability. The rate calculator described by Van Amburgh et al. (2003) was used to calculate the rate of NDF degradation.

### Statistical analysis

The study used multiple statistical approaches to analyze production, DMI, and rumen data, accommodating for repeated measurements, individual animal effects, and potential crossover design impacts where applicable. Statistical analyses were conducted using SAS software (Version 9.4; SAS Institute Inc., Cary, USA), with specific models tailored to the characteristics of each dataset. Differences were deemed significant if *P* ≤ 0.05, and 0.05 < *P* ≤ 0.10, would indicate a trend toward significance.

### Production data

Cows within the 2 treatments were randomly assigned to 17 block replicates in a randomized complete block design. Levene’s test confirmed the homogeneity of treatment variances (Levene, 1960), while the Shapiro–Wilk test (Shapiro & Wilk, 1965) validated the normality of standardized residuals. Although daily data were collected, mean values were calculated for each cow across the trial period to reflect cumulative performance, as the primary objective was to assess treatment-level differences rather than temporal trends. Data were analyzed using the General linear model (**GLM**) procedure of SAS software (Version 9.4; SAS Institute Inc., Cary, USA). The GLM procedure was chosen for its suitability with balanced data and straightforward modeling of treatment and blocking effects. Treatment was modeled as a fixed effect, while block and the residual error were treated as random effects. Least Square Means (**LS means**) were calculated to compare treatment effects.

### Dry matter intake data

A randomized complete block design with 10 replicates was used for the DMI study. Treatments were randomly assigned within each block. Levene’s test confirmed the homogeneity of treatment variances (Levene, 1960), while the Shapiro–Wilk test (Shapiro & Wilk, 1965) validated the normality of standardized residuals. Data were analyzed using the GLM procedure of SAS software (Version 9.4; SAS Institute Inc, Cary, USA), with treatment as a fixed effect and block and residual as random effects. Least Square Means were calculated to compare treatment effects.

### Rumen data

Six cows were randomly allocated to the 2 treatments in a 2-period crossover design with 2 sequences. Levene’s test confirmed the homogeneity of treatment variances (Levene, 1960), while the Shapiro–Wilk test (Shapiro & Wilk, 1965) validated the normality of standardized residuals. Data were analyzed using the GLM procedure of SAS software (Version 9.4; SAS Institute Inc, Cary, USA). Treatment and period were modeled as fixed effects, while cow nested within sequence and the residual error were treated as random effects. Least Square Means were calculated to compare treatment means.

## Results

### Feed and pasture

The chemical composition of the 2 concentrates and pasture is shown in **Table 3**. The 2 concentrates were formulated to have identical nutrient compositions; however, minor differences existed in the analyzed chemical compositions (**Table 3**). This was not expected, but could be due to sampling and analysis variation. Pasture had a ME value of 10.6 MJ.kg^-1^ DM, CP value of 163 g.kg^-1^ DM, and a NDF value of 494 g.kg^-1^ DM, falling within the recommended ranges for perennial ryegrass swards (Wims et al., 2010). Average pre-and post-grazing RPM heights were 30.9 and 11.6 RPM units, respectively (**Table 4**).

**Table 3.** Analyzed chemical composition of concentrates (*n* = 2) and pasture (*n* = 1) (g/kg DM) (mean ± SE) consumed by cows (*n* = 17) during the trial

Parameter, g.kg^-1^ DM^1^	Treatments^2^		Pasture
	Control	AOFP	
DM	908 ± 9.00	905 ± 1.53	169 ± 4.56
OM	928 ± 0.47	926 ± 2.40	890 ± 4.77
CP	154 ± 0.92	145 ± 1.43	163 ± 11.0
EE	36.0 ± 0.17	36.1 ± 0.44	45.6 ± 0.01
aNDF	165 ± 13.0	172 ± 8.15	494 ± 6.89
ADFom	75.1 ± 3.24	73.5 ± 4.94	342 ± 11.7
Lignin (sa)	2.11 ± 0.21	2.11 ± 0.21	10.1 ± 1.05
ME, MJ.kg^-1^ DM	12.2 ± 0.25	12.2 ± 0.01	10.6 ± 0.05
Calcium	11.7 ± 0.44	11.5 ± 0.28	3.65 ± 0.24
Phosphorus	4.41 ± 0.04	4.39 ± 0.08	3.87 ± 0.27
Magnesium	3.93 ± 0.11	3.83 ± 0.10	2.75 ± 0.15
Potassium	9.96 ± 0.22	9.29 ± 0.10	35.6 ± 3.40
Sodium	1.77 ± 0.10	1.81 ± 0.07	6.53 ± 1.39
Manganese	0.14 ± 0.003	0.13 ± 0.005	0.05 ± 0.006
Copper	0.03 ± 0.003	0.03 ± 0.003	0.005 ± 0.0004
Iron	0.20 ± 0.008	0.21 ± 0.021	0.12 ± 0.01
Zink	0.17 ± 0.002	0.17 ± 0.003	0.03 ± 0.003

^1^DM, Dry matter; OM, Organic matter; CP, Crude protein; EE, Ether extract; aNDF, Neutral detergent fiber assayed with heat-stable alpha-amylase, inclusive of residual ash; ADFom, Acid detergent fiber exclusive of residual ash; ME, Metabolizable energy.

^2^Control—6 kg.cow^-1^.d^-1^ standard dairy concentrate; AOFP—6 kg.cow^-1^.d^-1^ standard dairy concentrate containing 0.5 kg.ton^-1^*Aspergillus oryzae* fermentation product (AOFP).

**Table 4.** Average pre- and post-grazing pasture measurements, herbage yield, allowance, and intake (mean ± SD)

Parameter	Pasture values
**RPM height**	
Pre-grazing	30.9 ± 8.99
Post-grazing	11.6 ± 1.99
**Pasture yield, kg DM.ha** ^**-1**^	
Pre-grazing	2,926 ± 925.9
Post-grazing	939 ± 204.6
**Pasture removed, kg DM.ha** ^**-1**^	1,987 ± 825.86
**Daily herbage allowance, kg DM.cow** ^**-1**^	15.9 ± 2.97
**Daily pasture removed, kg DM.cow** ^**-1**^	10.7 ± 2.50

### Production study data

The body weight of cows in the control and AOFP treatments increased by 36.2 and 28.1 kg, respectively (**Table 5**), indicating that cows consumed sufficient feed to meet maintenance and production requirements. The body condition did not change from the start to the end of the trial, and there were no differences in body weight or condition between the 2 treatments before (*P* = 0.79) and after (*P* = 0.71) the trial.

**Table 5.** Production study data of cows (*n* = 17) grazing ryegrass-dominant pastures during spring with or without AOFP supplementation

Parameter	Treatment		SEM	P-value^1^
	Control	AOFP		
Milk yield, kg.cow^-1^.d^-1^	20.9	20.4	0.403	0.41
4% FCM, kg.cow^-1^.d^-1^	24.1	24.3	0.423	0.70
ECM, kg.cow^-1^.d^-1^	26.1	26.2	0.422	0.85
Milk fat content, g.kg^-1^	50.6	52.9	0.097	0.10
Milk protein content, g.kg^-1^	40.3	40.4	0.042	0.85
Milk lactose content, g.kg^-1^	47.8	48.0	0.036	0.74
MUN (mg.dL^-1^)	6.93	6.75	0.265	0.64
SCC (×1,000.mL^-1^)	116	105	32.65	0.81
BW before, kg	393	397	8.062	0.79
BW after, kg	430	425	9.233	0.71
Change in BW, kg	36.2	28.1	3.283	0.10
BCS before	2.27	2.27	0.015	1
BCS after	2.27	2.28	0.018	0.58
Change in BCS	0	0.02	0.018	0.58

BW, Body weight; BCS, Body condition score (scale 1 to 5); BC, Body condition; ECM, Energy corrected milk; FCM, Fat-corrected milk; MUN, Milk urea nitrogen; SCC, Somatic cell count; SEM, standard error of the mean.

^1^ *P*-value, *P* ≤ 0.05 = significant difference; *P* > 0.05 = no significant difference.

Milk yield (*P* = 0.41), 4% FCM yield (*P* = 0.70), and ECM yield (*P* = 0.85) did not differ between the 2 treatments (**Table 5**). There was a tendency (*P* = 0.10) for a 2.3 g.kg^-1^ greater milk fat content in cows on the AOFP treatment. Other milk composition parameters were unaffected (*P* > 0.05).

### Dry matter intake study data

The pasture DMI, total DMI, DMI as a percentage of body weight, and feed efficiency of cows in the control and AOFP treatment are presented in **Table 6**. The average daily pasture (*P* = 0.17) and total DMI (*P* = 0.17) did not differ between treatments. The daily pasture intake was 7.42 kg per cow for cows in control and 8.78 kg per cow for cows on the AOFP treatment. Total daily DMI was 12.9 kg per cow for cows in the control, and 14.2 kg per cow for cows on AOFP treatment. Feed efficiency (kg ECM per kg DMI) was 2.03 and 1.82 for cows on the control and AOFP treatment, respectively, with no differences observed between treatments (*P* > 0.05).

**Table 6.** The dry matter intake of Jersey cows (*n* = 10) grazing ryegrass-dominant pasture during Spring.

Parameter	Treatment		SEM	*P*-value^1^
	Control	AOFP		
Daily fecal output, kg.cow^-1^	2.98	2.92	0.152	0.79
Daily pasture DMI, kg.cow^-1^	7.42	8.78	0.642	0.17
Daily total DMI, kg.cow^-1^	12.9	14.2	0.642	0.17
DMI as % BW	2.97	3.33	0.166	0.16
Feed efficiency	2.03	1.82	0.135	0.29

BW, Body weight; DMI, Dry matter intake; Feed efficiency, Calculated as a ratio between energy corrected milk and total DMI; SEM, standard error of the mean.

^1^ *P*-value, *P* ≤ 0.05 = significant difference; *P* > 0.05 = no significant difference.

### Rumen study data

**Figure 3.**
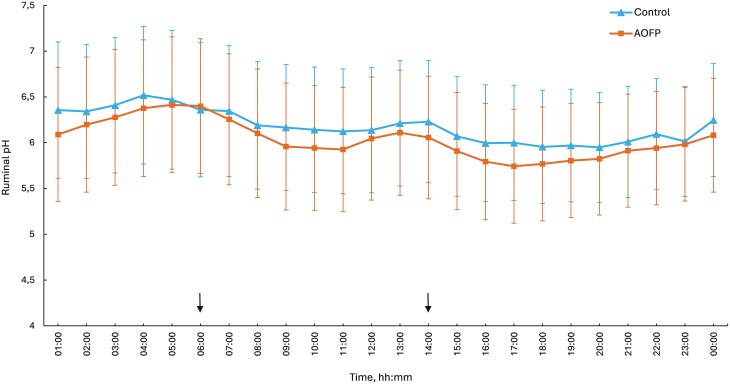
Diurnal fluctuations in ruminal pH of cows (*n* = 6) grazing ryegrass-dominant pasture in spring, with or without AOFP supplementation. Error bars represent the SEM.

**Figure 3** presents the diurnal pH fluctuations in rumen-fistulated cows measured using a TruTrack pH data logger. The black arrows on the graph indicate the times of milking and concentrate consumption. A distinct decline in rumen pH after concentrate consumption can be seen. Rumen pH reached its lowest point in the afternoon post-milking, and recovered after 20:00, consistent across both treatments. Cows on the AOFP treatment exhibited a pH below 6.2 for a longer time than cows in the control treatment (*P* = 0.05; **Table 7**). The average rumen pH of cows on the AOFP treatment was also lower (*P* = 0.05) than cows in the control treatment, at pH 6.18 and pH 6.10, respectively.

**Table 7.** Average ruminal pH values measured with a TruTrack data logger and time spent below a specific pH (5.8; 6; 6.2) in Jersey cows (*n* = 6) grazing ryegrass-dominant pasture with or without AOFP supplementation

Parameter^1^	Treatment		SEM^2^	*P*-value^3^
	Control	AOFP		
**Average pH (24 h)**	6.18	6.10	0.222	0.05
**pH 1 hour after milking**				
07:30	6.30	6.28	0.037	0.70
15:30	6.05	5.94	0.042	0.14
**Time below (h)**				
pH 5.8	1.50	4.75	1.630	0.23
pH 6	4.08	8.33	1.275	0.08
pH 6.2	9.45	13.3	0.969	0.05

^1^Time below—the hours that the pH was below a certain level in 24 h.

^2^SEM, standard error of the mean.

^3^ *P*-value, *P* ≤ 0.05 = significant difference; *P* > 0.05 = no significant difference.

Rumen VFA and ammonia-nitrogen concentrations are presented in **Table 8**. Treatment did not affect total VFA concentrations (*P* = 0.98) or the acetate-to-propionate ratio (*P* = 0.12). Rumen ammonia nitrogen concentrations did not differ between treatments (*P* = 0.18), with cows in the control treatment having a NH_3_-N concentration of 11.4 mg.dL^-1^ and cows on the AOFP treatment a NH_3_-N concentration of 9.94 mg.dL^-1^.

**Table 8.** Rumen study data of rumen cannulated Jersey cows (*n* = 6) with or without AOFP supplementation

Parameter	Treatment		SEM	*P*-value^1^
	Control	AOFP		
Total VFA, mmol.L^-1^	111.21	111.32	3.721	0.98
Acetate: Propionate	4.45	4.24	0.077	0.12
Acetate, mmol.L^-1^	80.96	80.34	2.570	0.87
Propionate, mmol.L^-1^	18.28	19.06	0.832	0.55
Butyrate, mmol.L^-1^	9.07	8.94	0.198	0.66
Isobutyrate, mmol.L^-1^	0.89	0.87	0.064	0.81
Valerate, mmol.L^-1^	1.35	1.46	0.072	0.35
Iso-Valerate, mmol.L^-1^	0.66	0.65	0.038	0.93
NH_3_-N, mg.dL^-1^	11.4	9.94	0.631	0.18
**DMd, g.kg** ^**-1**^				
6 h	479	481	10.6	0.91
18 h	665	668	16.3	0.90
30 h	815	814	5.10	0.85
**NDFd, g.kg** ^**-1**^				
6 h	231	242	17.1	0.62
18 h	482	493	23.0	0.77
30 h	708	705	9.10	0.82
**NDF k**_**d**_, **(%.h**^**-1**^)	4.36	4.39	1.48	0.88

VFA, Volatile fatty acids; NH_3_-N, Ammonia Nitrogen; DMd, Dry matter disappearance; NDFd, Neutral Detergent Fiber disappearance; NDF k_d_^2^, Rate of NDF disappearance (%.h^-1^); SEM, standard error of the mean.

^1^ *P*-value—*P* ≤ 0.05 = significant difference; *P* > 0.05 = no significant difference.

Dry matter and NDF degradation after 6, 18, and 30, and the rate of NDF disappearance (**NDFd k**_**d**_^**2**^) did not differ (*P* > 0.05) between treatments (**Table 8**).

## Discussion

Feed additives have successfully been used in livestock production systems to increase nutrient intake and utilization, and boost production efficiency (Vieco-Saiz et al., 2019) and are particularly beneficial during periods of physiological stress (El Jeni et al., 2024; Kholif et al., 2024). The high NDF value of the pasture (494 g.kg^-1^ DM) and the low levels of concentrate supplementation did not pose a risk to rumen pH stability or overall rumen health, which may have limited the potential impact of AOFP in the cows.

Considering previous studies performed on the Outeniqua Research Farm (Van der Colf et al., 2015; Van Wyngaard et al., 2019), the pasture CP value was lower than expected. Milk urea nitrogen was also lower than the recommended values (10 and 14 mg. dL^-1^), but with a milk yield of 20 L in cows on both treatments, the total dietary protein was sufficient to meet production requirements (NRC, 2021). This is further supported by acceptable rumen NH_3_-N and milk protein levels (Wilkinson et al., 2020).

Cows in both treatment groups grazed together, ensuring equal pasture access. Pasture management aimed to balance intake potential with optimal pasture utilization. The average post-grazing sward height of 11.6 RPM units, and daily herbage allowance of 15.9 kg DM per cow per day, suggest sufficient pasture availability, and pasture management was satisfactory (Irivine et al., 2010). Allocating more pasture may have increased intake slightly, however, increased herbage allowance surpassing the point of efficient utilization by the cows will increase the refusal of pasture, leading to wastage and shadowing of the growing points, reducing the nutritive value of the pasture in future grazing cycles (McEvoy et al., 2009; Méndez et al., 2020; Wilkinson et al., 2020). Pasture intake is primarily determined by rumen capacity and the rate at which the rumen is emptied, both of which are influenced by the chemical composition and degradability of the pasture (Jung & Allen, 1995; Wilkinson et al., 2020). Pasture degradability is linked to its neutral detergent fiber (NDF) content and the degradability of the NDF. The relatively high pasture NDF content in this study may have contributed to rumen fill limitations, restricting voluntary intake. However, given the milk production of approximately 24 kg of 4% FCM, achieved with only 6 kg of concentrate fed daily, alongside increases in body weight, suggests cows were generally well fed. Cows consumed 3% to 3.3 % of their body weight as DMI, which is consistent with findings reported by Bangani et al. (2023) for grazing Jersey cows. In contrast to a study conducted by Caton et al. (1993), supplementing AOFP did not alter pasture or total DMI. Given that AOFP did not affect DMd and NDFd, a response in DMI was not expected. This is consistent with the similar milk yield of cows in both treatments. Other studies conducted in TMR and in vitro systems reported increased DM and NDF degradation (Chen et al., 2004; Sun et al., 2013). The rate of NDF degradation (4.36%.h^-1^ and 4.39%.h^-1^ for the control cows and AOFP-supplemented cows) was within the 2% to 9%.h^-1^ range reported by Hoffman et al. (1993) for in situ NDF degradation of ryegrass. When AOFP was supplemented to heifers on high-quality pasture, average daily gain was improved (Gray et al., 2013), indicating that under different grazing conditions (poorer or higher quality pasture), the response to AOFP supplementation may differ. Cows supplemented with AOFP had a lower rumen pH for a longer duration than cows in the control treatment, but remained within the optimal range for proper rumen function (Bargo et al., 2003). Lower rumen pH indicates higher ruminal fermentation, but rumen VFA concentrations were similar between cows in the respective treatments. The tendency for a 2.3 g.kg^-1^ greater milk fat content in the AOFP treatment was not supported by the rumen study data. Previous researchers reported greater milk fat content when AOFP was supplemented (Bach, 2003; Baumgard, 2004), whereas others reported no change in milk fat content (Kellems et al., 1990; Gomez-Alarcon et al., 1991; Denigan et al., 1992; Sievert & Shaver, 1993; Higginbotham et al., 2004; Sun et al., 2013; Zhang et al., 2022). Individual cow variation complicates the prediction of feed additive effects on milk composition.

In summary, the supplementation of AOFP at 3 g.cow^-1^.d^-1^ to Jersey cows grazing perennial ryegrass pasture had limited effects on milk production and composition, and rumen parameters in this study. The effect of AOFP on dairy cows in previous literature has conflicting results. This suggests that it may vary depending on the type of system (TMR or pasture-based), pasture type and quality, the level of supplementary feeding (concentrate), and the composition of the concentrate. The AOFP dosage used in this study was based on the manufacturer’s recommendation for lactating dairy cows and aligns with standard commercial feeding practices. While this rate is commonly applied in TMR-based systems, its efficacy under pasture-based conditions remains less explored. It is therefore possible that a higher inclusion rate could elicit different outcomes, but would need to be evaluated with consideration for practical feasibility and cost-effectiveness on pasture-based farms. Future research should investigate the effects of AOFP supplementation on pasture-based dairy cows across different pasture types and qualities, varying levels of supplementary feeding and dietary compositions, as well as determine the dose–response relationship of AOFP under these grazing conditions. The impact of AOFP beyond the rumen should also be explored in future studies.

## Abbreviations

ADF: acid detergent fiber
AOFP: *Aspergillus oryzae* fermentation product
BCS: body condition score
CP: crude protein
DM: dry matter
FCM: fat-corrected milk
ECM: energy-corrected milk
EE: ether extract
ME: metabolizable energy
MUN: milk urea nitrogen
NDF: neutral detergent fiber
NDF k_d_^2^: rate of neutral detergent fiber degradation
NH_3_-N: ammonia nitrogen
OM: organic matter
SCC: somatic cell count
TMR: total mixed ration
VFA: volatile fatty acids
